# Different Technical Applications of Carbon Nanotubes

**DOI:** 10.1186/s11671-015-1056-3

**Published:** 2015-09-16

**Authors:** S. Abdalla, F. Al-Marzouki, Ahmed A. Al-Ghamdi, A. Abdel-Daiem

**Affiliations:** Department of Physics, Faculty of Science, King Abdulaziz University Jeddah, P.O. Box 80203, Jeddah, 21589 Saudi Arabia

**Keywords:** Carbon nanotubes, CVD, Microelectronics, Applications

## Abstract

Carbon nanotubes have been of great interest because of their simplicity and ease of synthesis. The novel properties of nanostructured carbon nanotubes such as high surface area, good stiffness, and resilience have been explored in many engineering applications. Research on carbon nanotubes have shown the application in the field of energy storage, hydrogen storage, electrochemical supercapacitor, field-emitting devices, transistors, nanoprobes and sensors, composite material, templates, etc. For commercial applications, large quantities and high purity of carbon nanotubes are needed. Different types of carbon nanotubes can be synthesized in various ways. The most common techniques currently practiced are arc discharge, laser ablation, and chemical vapor deposition and flame synthesis. The purification of CNTs is carried out using various techniques mainly oxidation, acid treatment, annealing, sonication, filtering chemical functionalization, etc. However, high-purity purification techniques still have to be developed. Real applications are still under development. This paper addresses the current research on the challenges that are associated with synthesis methods, purification methods, and dispersion and toxicity of CNTs within the scope of different engineering applications, energy, and environmental impact.

## Introduction

**Fig. 1:**
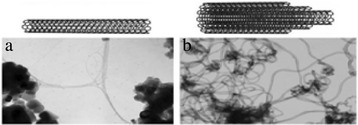
Molecular representations of SWCNT (**a**) and MWCNT (**b**) with typical TEM [9]

Carbon nanotubes (CNTs) emerged in the field of nanotechnology because of their nanosize and unique properties. Carbon nanotubes are hollow cylinders made of graphite carbon atoms with nanoscale (10^−9^ m) which is much smaller than the human hair width [1]. These CNTs are the members of fullerene structural family with their ends capped with a hemisphere of bucky ball structure [2]. CNTs have a broad range of electronic, thermal, and structural properties because of their nanosize which may vary with their length, diameter, and chirality [3]. CNTs show high values of thermal conductivity [4–6], Young’s modulus [1, 7], large surface area [4], high current density [1, 7], ballistic transport on submicron scales, and massless Dirac fermion charge carrier abilities [4] which make their ability in the wide applications such as photovoltaic devices, sensors, transparent electrodes, supercapacitors, and conducting composites [4]. Due to their unique properties, their synthesis, purification, and applications, CNTs have become an attractive and interesting research area in the field of nanotechnology [1]. It has been proved that instead of using the as-synthesized CNTs, if their surfaces are modified or functionalized with chemical compounds, it increases their properties in the usage of electrolytes, biology, and energy storage [8]. Carbon nanotubes are categorized as multi-walled nanotubes (MWNTs) or single-walled nanotubes (SWNTs) [2]. SWNT in Fig. 1a is a single-rolled graphene and MWNT in Fig. 1b is a multi-rolled graphene. This paper gives an overview of the composite carbon nanotube application and their future challenges which provides the information for further research.

## Review

**Fig. 2:**
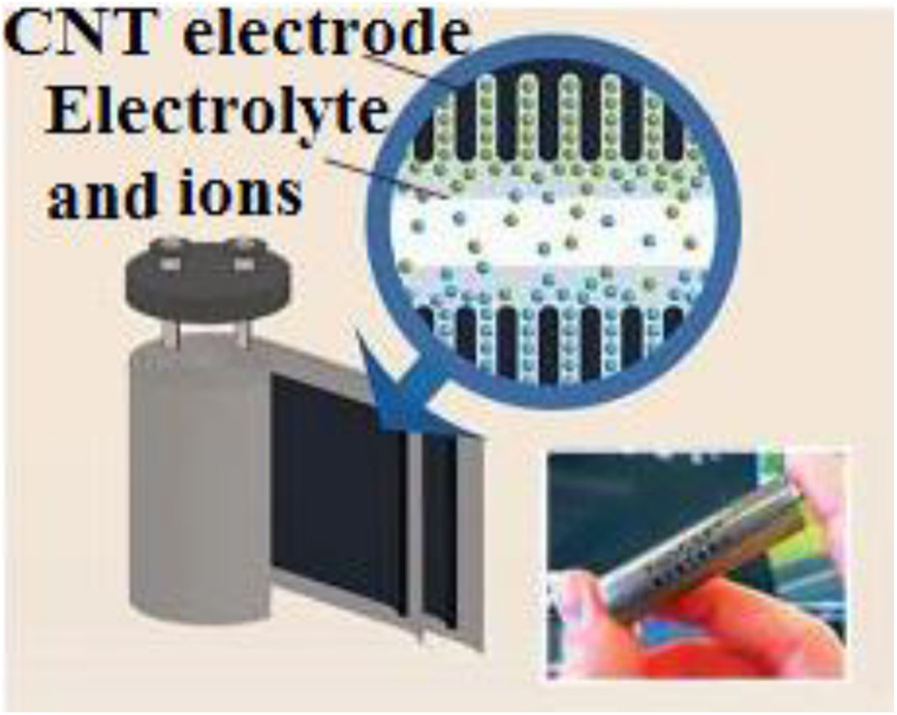
Supercapacitor based on CNTs [17]

**Fig. 3:**
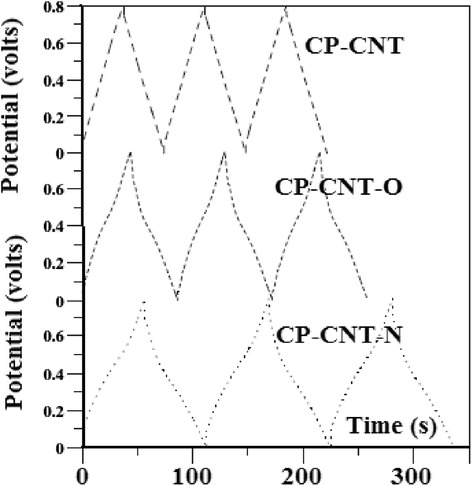
Charge–discharge curves [8]

**Fig. 4:**
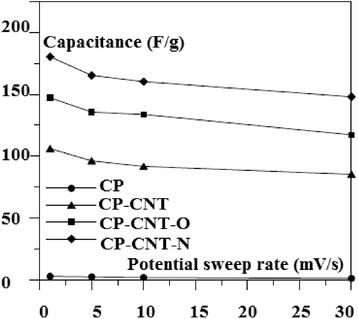
Variation of specific capacitance [8]

**Fig. 5:**
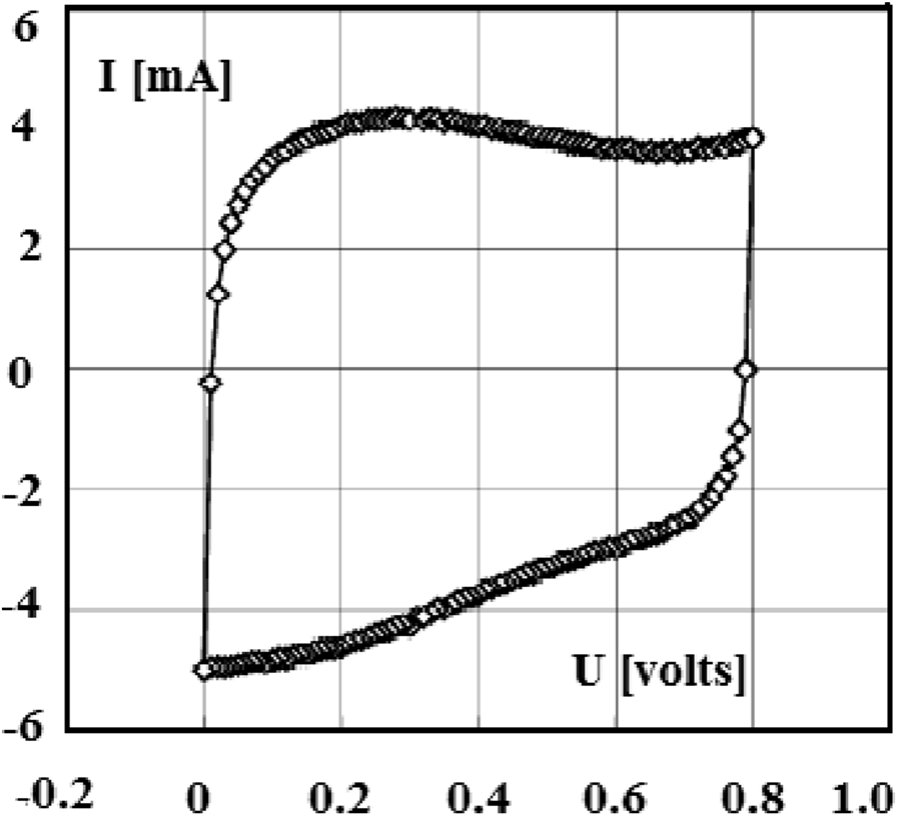
Voltammetry characteristics (10 mV/s) [18]

Carbon nanotubes have unique properties because of their structure and size which will be discussed in this section. Several recent researches are now being developed using carbon nanotubes (CNTs), for example, using CNTs as electrical electrodes for the brain researches [9, 10] where the scientists have bundle millions of CNTs into micron-sized threads. Moreover, CNTs have been combined with indium–gallium zinc oxide (In-Ga-ZnO (IGZO)) to build a more efficient hybrid computer chip which is more transparent, flexible, and more energy saving (efficient) than the typical silicon chips. Also, some future applications include nanoelectronic circuits, memory chips, organic light-emitting diodes, and different sensors. This may lead to production of wearable, flexible computers. This is the axis that the computing manufacturing is transferring towards. Recently, we have shown different applications concerning CNTs [11–14] as we will see later. SWNTs are believed to possess strong mechanical properties due to strong covalent bond among the carbon atoms [1] (Tables 1 and 2). They have Young’s modulus of 1 TPa [15] which is five times stiffness than steel [16]. They are highly flexible and attain their original shape after buckling and bending [17] and have low density of about one sixth of the density of steel [1]. Moreover, the property of carbon nanotube being insulator, semiconductor, or conductor depends on the chirality, the way in which carbon atoms are arranged [1]. In addition, below room temperatures and above 1 K, the specific heat and thermal conductivity are measured by phonons. It has been reported that the specific heat and thermal conductivity are linearly dependent on temperature [5]. Carbon nanotubes possess high thermal conductivity in the axial direction and found to be twice that of the diamond [1, 5]. For the different techniques that authors have used at low cost in large-scale and continuous production, we summarize the following: arc-discharge method, where it was the first method to produce nanotubes by the arc vaporization of two graphite rods which are placed in an inert gas for the reaction. Carbon rods are evaporated by direct current which will create high temperature discharge between two electrodes, due to which anode gets evaporated and nanotubes will be deposited on cathode [3]. Second is laser ablation method, where a piece of graphite is vaporized by laser irradiation under an inert atmosphere. This results in soot containing CNTs which are cooled at the walls of a quartz tube. This method is very expensive, so it is mainly used for SWNTs [3]. High-purity SWNTs have been produced with less defects and contaminates [15]. Third is chemical vapor deposition method; in this method, the nanotubes are produced by the thermal decomposition of hydrocarbon in the presence of metal catalyst [15]. Chemical vapor deposition (CVD) is the simple and economic technique to synthesize CNTs at relatively low temperature, but with less purity. However, the patterning design of catalyst in CVD technique has controlled the size, shape and alignment of the nanotube [17]. Fluidized bed chemical vapor deposition is an efficient technique to deposit on, to functionalize, or to coat each individual particle of a powder from gaseous species. This technique joins two processes on the same time: The first is the deposition process and the second is resisting the particles in the deposition area by introducing a gas upwards through the powder. Due to their very high growth surface to heated volume ratios (S/V), fluidized bed chemical vapor deposition has limited applications. However, this technique is versatile and has high gas conversion, possibility of continuous operations, good homogeneity of products, technology easy to use and to rescale, and low costs in equipment. Moreover, this technique is suitable to perform discontinuous deposits on non-porous powders, as to treat porous. The real challenge nowadays is due to characterizations of conditions ensuring high deposition rates to carry out thick continuous deposits on non-porous powders, because clogging phenomena and parasitic fine particle formation can occur. These processes are relative to the mastery of fluidization quality for either lowly fluidizable initial powders or particles of evolutionary morphology due to the deposit [18]. Moreover, the concept of supercapacitor based on CNTs is demonstrated in Fig. 2. Because of high capacitance, supercapacitors are widely used in electronic devices. Nanotubes are used as electrodes in supercapacitors, in which the capacity measured is inversely proportional to the separation between the electrode and electrolyte interface which is less for the nanotubes [19] (Table 3). These electrochemical capacitors are believed to possess high power density and long durability without short circuit, which makes their application more interesting in the research area [17]. Their properties are still improved by using functionalized CNT electrodes, which are discussed briefly. The experiments [8] on the electrochemical capacitor with functionalized CNTs in H_2_SO_4_ electrolyte solution have been studied. It has been observed that water molecules and the presence of surface oxides/amides (in case of CP–CNT–O and CP–CNT–N) on CNT surface [20] may cause hindrance to the migration of electrolytes and could be the reason to show up high ohmic resistance, which results in delay in charge and discharge cycle as presented in Fig. 3. It has been reported that amino groups on the functionalized CNTs [21] are capable of participating in the redox process of amine moieties, due to which it plays a major role as a contributor because of the electron-donating capability of the electron-rich phenyl ring. The electrical conductivity of the carbon nanotubes is 1000 times higher than that of the copper [22]. The investigation of electrochemical characteristics [23] has been carried out with MWNTs. It is reported that the capacitance value of carbon nanotubes was as high as 80 F/g with moderate surface area 450 m^2^/g (Fig. 4). But when MWNTs were functionalized with nitric acid, their capacitance value was increased to 130 F/g and the reversible ill-defined redox peak was observed at 0.2 V in voltammetry curve in Fig. 5 due to surface functionality by oxygen. Polypyrrole (PPy) was deposited on the MWNTs, which have been used as electrodes in the supercapacitors. Their capacitance was determined by performing cyclic voltammetry and charge–discharge cycling. Further observed was the effect of PPy on MWNTs by voltammetry, in which the square shape voltammogram relates the best combination between PPy and MWNTs, which was confirmed by the linear discharge on the galvanostatic curve. The high values of capacitance were due to the opened mesopore network in the composite nanotubes and the involvement of the thin layer of bulk PPy in the pseudo Faradic processes [24].

**Table 1:** Mechanical properties of different materials for comparison of carbon nanotubes [16]

Density (g/cm^3^)	Tensile strength (GPa)	Young’s modulus (GPa)	Material
~0.7–1.7	~100–200	~1000	SWNT/MWNT
7.8	1.3	210	High tensile steel
1.75	3.5	230	Toray carbon fibers
1.44	3.6	60	Kelvar
2.6	3.4	22	Glass fibers

**Table 2:** Transport properties of conductive materials [16]

Electrical conductivity	Thermal conductivity (W/m k)	Material
10^6^–10^7^	>3000	Carbon nanotubes
6 × 10^7^	400	Copper
2–8.5 × 10^6^	1000	Carbon fiber–pitch
6.5–14 × 10^6^	8–105	Carbon fiber–PAN

**Table 3:** Some of the reported storage capacity of hydrogen in CNTs

Reference	Stored hydrogen (wt%)	Discharge capacity	Material
Nutzenadel et al. [45]	0.39	110	SWNT
Rajalakshmi et al. [46]	2.9	800	SWNT
Gao et al. [47]	1.051	297	MWNT
Frackowaiak et al. [24]	<0.5	<141	SWNT
Dai et al. [48]	1.84	503	Aligned SWNT

**Fig. 6:**
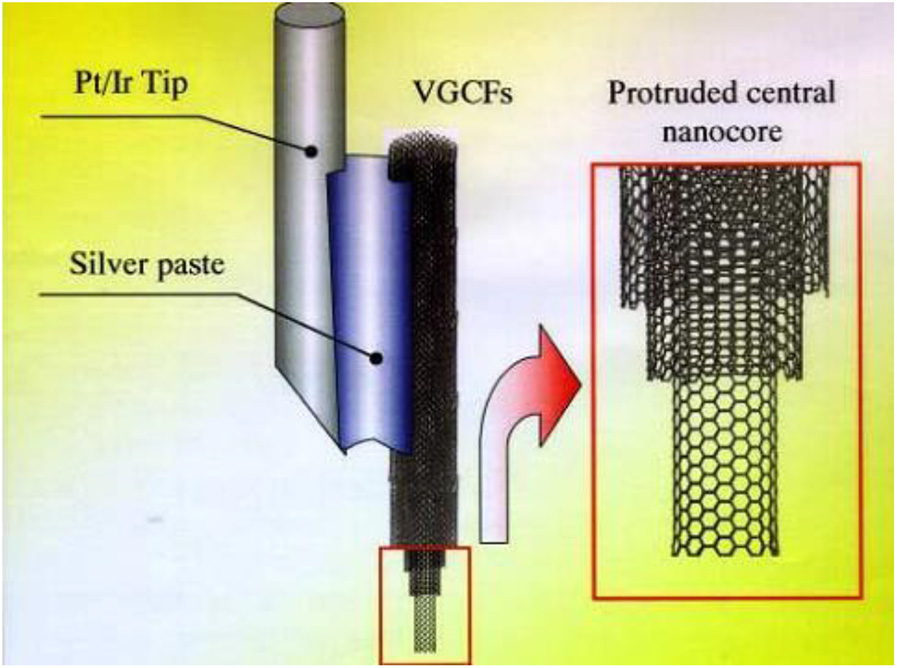
Use of CNTs as AFM tip [25]

Also, the unique properties of nanotube with extremely small size, high conductivity, and high mechanical strength have become paramount in nanoprobe application. Such probes can be used in various applications such as drug delivery, nanoelectrodes, high-resolution imaging, sensors, and field emitting device. As the CNT electrodes are electrically conductors, this permits them to be used in STM, AFM instrument as well as in an electrostatic force microscope [25]. Figure 6 demonstrates the use of CNT attached to the end of the SEM tip. Using the nanotube tips, the biological molecules can be viewed (by their image) with high resolution, and also by using the tips in tapping mode, the amyloid-b-protofibril biological molecules can also be imaged [26]. The high elasticity property of the nanotube prevents the tips from suffering mechanical destruction on contact with the substrate, and any impact on the nanotube will cause buckling which is reversible on detachment of tip from the substrate. Apart from using nanotube tip for high-resolution imaging, they can also be used as an active tool for surface manipulation. The nanotube can be used like a pair of tweezers to get rid of nanoscale structures on the surface if the pair of nanotubes can be localized on the AFM tip correctly [27]. The nanotube can also be used as molecular probes with its application in chemistry and biology by chemically modifying the nanotube tip through the attachment of functional groups [28]. Nanotubes can, also, be used for chemical and biological discrimination on surfaces [29] when using the attachment of acidic functionalities. The functionalized nanotube tip can be used to estimate the binding forces between protein–ligand pairs. The nanotube can be used as chemical and biosensor. The electrical resistivity of CNTs changes on exposure to gaseous environment containing NO_2_ and NH_3_ and to some inert gases like He, N_2_, Ar, O_2_, and CO_2_ [30]. The presence of gases can be precisely detected by the change in conductance of CNTs. The responses of nanotube sensors [31] are much faster than the other conventional sensors.

**Fig. 7:**
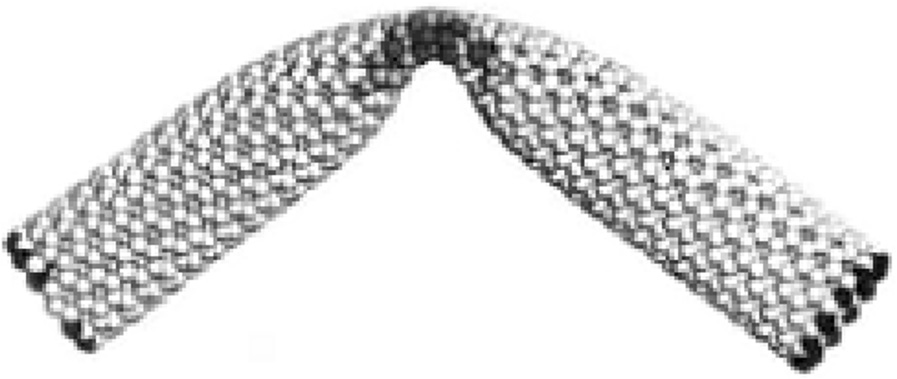
Flexible property of CNT [25]

**Fig. 8:**
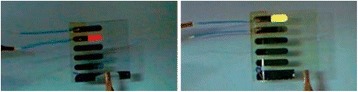
Application of CNT in OLED [25]

The mechanical property of carbon nanotubes is excellent for load-bearing reinforcements in composites and also for structural applications. The published data on nanotubes have revealed that they have some stiffest structures due to the carbon–carbon bond and the system controlling these atomic bonds throughout the axis of the nanotubes [32, 33]. Studies on SWNTs reveal that their Young’s modulus is about 1 TPa [34]. The observed tensile power of the tubes (using a nanostressing located within SEM) is slightly less than 60 GPa [35]. The very fascinating nature of CNTs is their fracture and deformation behavior. The simulation studies on SWNTs have observed the nanotubes to be highly deformed and seen to switch reversibly into different morphology patterns. On strain deformation, the CNTs get flattened, twisted, and buckled. They also tolerate high loads without showing the sign of fracture as shown in Fig. 7 [32]. Under TEM observation, the reversibility deformation for MWNTs has been recorded [36]. The plastic behavior of the nanotube has been projected recently [37]. The molecular dynamics simulation study predicts the fracture behavior of the nanotube under tensile loading at high temperature [38]. To increase the stiffness, strength, and toughness, the CNTs are mixed with polymers or resins for load-bearing applications [39]. Depending on the mechanical behavior, the use of nanotubes as reinforcements in composite materials is one of the most important applications of nanotubes. This application has limitations due to the improper interface between the nanotube and the polymer matrix to attain a good load transfer between the composite during loading [40–42]. Apart from the use in reinforcement, the nanotubes are also used for structural polymer composites. The toughness of the composite will increase with nanotube reinforcement by absorbing energy [43]. With non-linear optical and optical limiting properties, the nanotube–polymer systems are widely used for optical applications, including photovoltaic application [44]. In photovoltaic applications, the whole transport mechanism is altered by the use of functionalized and doped nanotubes in a photoactive polymer. In organic light-emitting diode (OLED), the addition of CNTs in a polymer system tunes the emission color as demonstrated in Fig. 8 [31]. These nanotube–polymer composites are also widely explored in electromagnetic induction (EMI) shielding application [45]. With their extremely small pore size, nanotubes are extensively used as membranes for molecular separation.

The application of nanotubes in hydrogen storage is due to their nanosize as well as cylindrical structure. Because of the capillary effect of nanotubes, it makes the storage of liquid or gas easier [19]. In order to get activated CNTs, one should use hydrogen storage materials with reproducible adsorption coefficients; this has not yet been realized. This is because hydrogen storage is only optimized for a very limited distribution of CNTs of distinct radius and types. However, several studies assume that a range of 4–14 wt% hydrogen adsorptions in CNTs is realizable; however, these studies did not distinguish between chemical and physical nature of absorption [19]. In interstitial sites of the host lattice in metal hydrides, hydrogen is stored reversibly. It was reported that in alkali metal intercalated CNT the hydrogen of 4–20 wt% is possible [31].

A photovoltaic process is a method of generating electrical power by converting solar radiation into direct current using a semiconductor. It has been stated that when polymers are combined with high-conductivity nanotubes, they disperse the nanotubes on the photoactive layer to obtain more efficient photovoltaic devices [46–50]. One can engineer the electronic structure with a nanophase of carbon, such as CNTs, and thus mobility can also be modified. This is applicable in photovoltaic devices. These devices can give crystalline exit pathways for charge transfer in organic solar cells when they are used as acceptor materials [51]. It was observed that for a high value of the photocurrent, sufficient interface for efficient exciton dissociation and continuous conducting paths for electrons and holes to the appropriate electrodes was responsible [52]. Considerable studies have been done to increase the efficiency of cell with composite nanotube as tabulated in Table 4 [53–56].

**Table 4:** Reported photovoltaic characteristics of the cell

Reference	Power efficiency (%)	Fill factor (%)	Short-circuit current density (μA/cm^2^)	Open-circuit voltage (V)	Source of illumination	Material used with SWNT
Adnen Ltaief et al. [53]	–	43	1	0.4	–	Poly(2-methoxy-5-(2′-ethyl hexyloxy)1-4-phenylene vinylene) (MEH-PV)
Kymakis et al. [54]	–	29	1	0.55	White light	Napthalocyanine in polyoctyl thiophene
Raffaella et al. [55]	–	26	–	0.85	Air mass zero	Poly(3-octyl thiophene)
Derbal-Habak et al. [56]	2.85	47.2	9560	0.670	107 mW/cm^2^	Poly(3-hexylthiophene): methanofullerene phenyl-C61-butyric acid methyl ester: before heat treatment
Derbal-Habak et al. [56]	3.66	52.2	11,240	0.668	107 mW/cm^2^	Poly(3-hexylthiophene): methanofullerene phenyl-C61-butyric acid methyl ester: after heat treatment

**Fig. 9:**
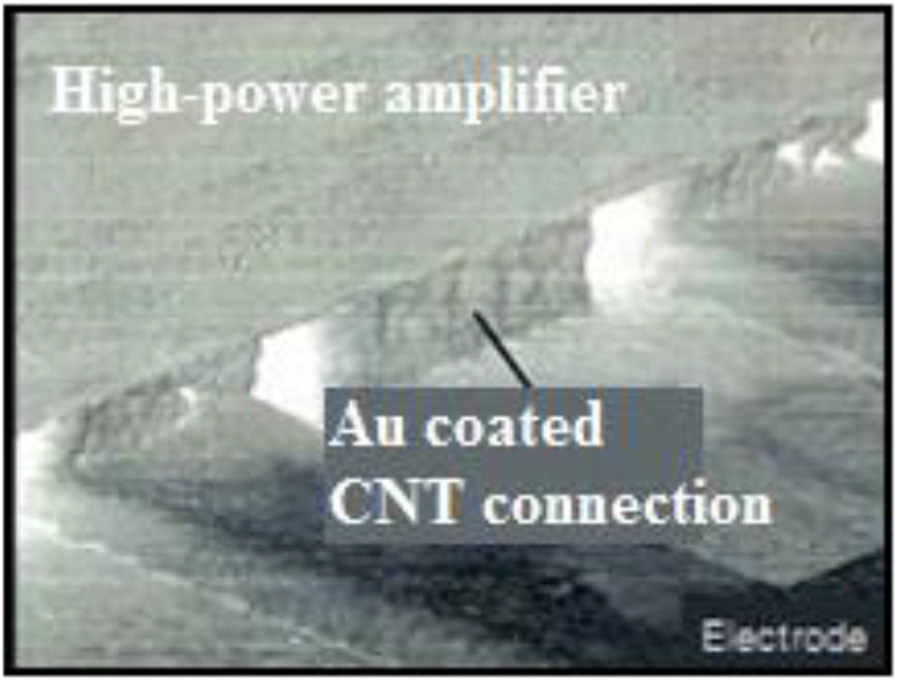
High-power amplifier [17]

**Fig. 10:**
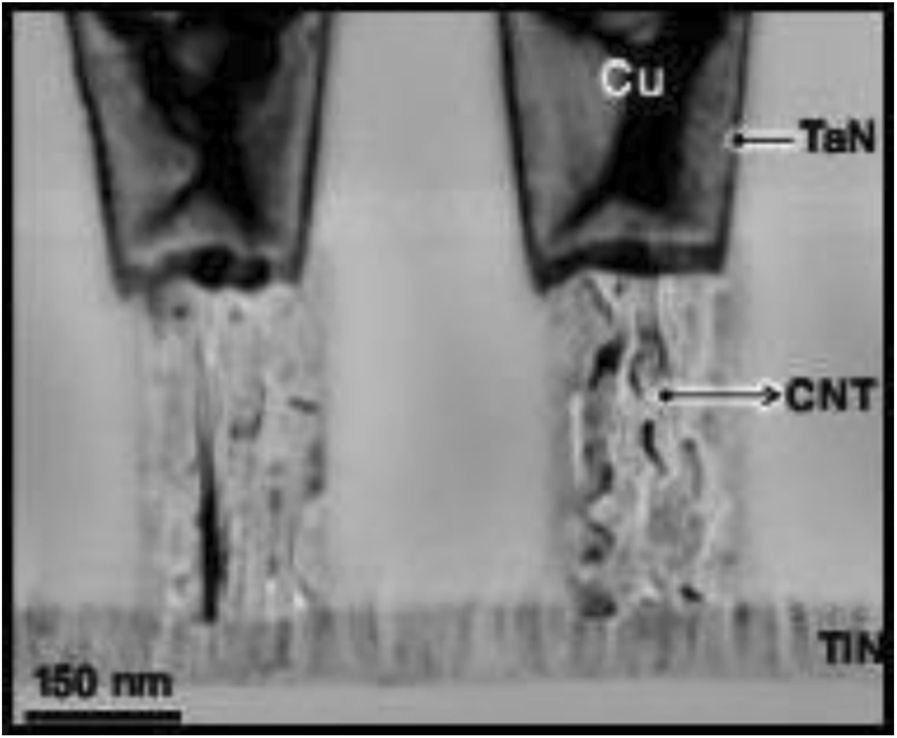
Electronic interconnect [17]

Because of low electron scattering and the suitable band gap, the SWNTs are attractive for transistor applications. The SWNTs are also well suited for field effect transistor (FET) architectures and high *κ* dielectrics [57]. Experimental results show the current density 2.41 mA/μm at 0.5 V for SWNT FETs, which is greater than those from silicon devices [58]. Even with excellent performance of SWNTs, they are still limited in microelectronic application due insufficient information regarding the control of CNT diameter, chirality, and density. SWNTs have achieved higher current densities and longer lifetime than the MWNTs or CNTs synthesized from CVD materials [59]. The SWNTs produced from laser ablation shows the current density of about 4 mA/cm^2^ [60]. The high mobility and low-temperature deposition of CNT thin-film transistors are widely used for OLED displays [61]. The sufficient current output to drive OLEDs at low voltage is shown by vertically aligned CNT FET, enabling the light emission by the OLED through a CNT network [62]. Low-cost printing of TFTs [63] and radio-frequency identification tags [64] are factors that promise growth of electronic CNTs. The excellent combination of properties of CNTs like nanosize diameter, high electrical conductivity, chemical stability, and integrated structures makes the fabulous electron emitters [31]. CNTs can also be used as electrical leads and heat dissipaters in high-power amplifier shown in Fig. 9 replacing the solder bumps and also as electronic interconnect as shown in Fig. 10. The more precise method for the development of high-density CNT film deposition method enables the fabrication of more CNTs in a single chip [65].

Being antioxidant in nature is the excellent property of carbon nanotube. This enhances its application in preservatives. To prevent the drug formulation and anti-aging cosmetics from oxidation, the nanotubes are generally used as preservatives [3]. To improve the rate of reaction, the carbon nanotube can be used as catalyst. As the nanotube has a large surface area, the catalyst can be incorporated into the nanotube and can be released in the desired quantity at specific time [3].

The fascinating application of CNTs is water purification. The tangled sheets of CNTs provide the excellent network with controlled nanoscale porosity, which electrochemically oxidize the organic contaminants [66], viruses, and bacteria [67]. For the purification of water, the filter containing CNTs have ben commercialized. The use of aligned CNTs with open ends in membranes allows the flow through the interior of CNTs [68]. This reduces the energy cost for water desalination by reverse osmosis in comparison to the commercial filtration by enhancing the permeability. In addition, the very small diameter of CNTs is required to discard the salt at sea water concentration [69]. The applications of nanotubes are related to their manufacturing processes and types of purification which assumes to be the major challenge for the nanotubes. It has been proved that MWNTs is a major contributor due to their availability in large quantity and also due to their certain properties which have specific application [70]. But single-walled carbon nanotubes have unique properties that are superior to those of multi-walled carbon nanotubes, especially in strength and electrical conductivity [1]. Because of their unique properties, SWNTs play a major role in the mechanical and electrical applications, where their strength can be improved by the development of composites on nanotubes or in polymer-based matrices [71]. As the growth mechanism of CNTs is not well understood, it is difficult to grow controlled CNTs so that their properties do not vary significantly with change in variability. In electronic applications, the chirality of CNTs becomes the important factor which enhances the electrical conductivity depending on the arrangement of atoms [72].

**Fig. 11:**
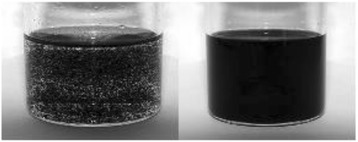
Example of poor (*left*) and good (*right*) dispersion of carbon nanomaterials in solution [64]

**Fig. 12:**
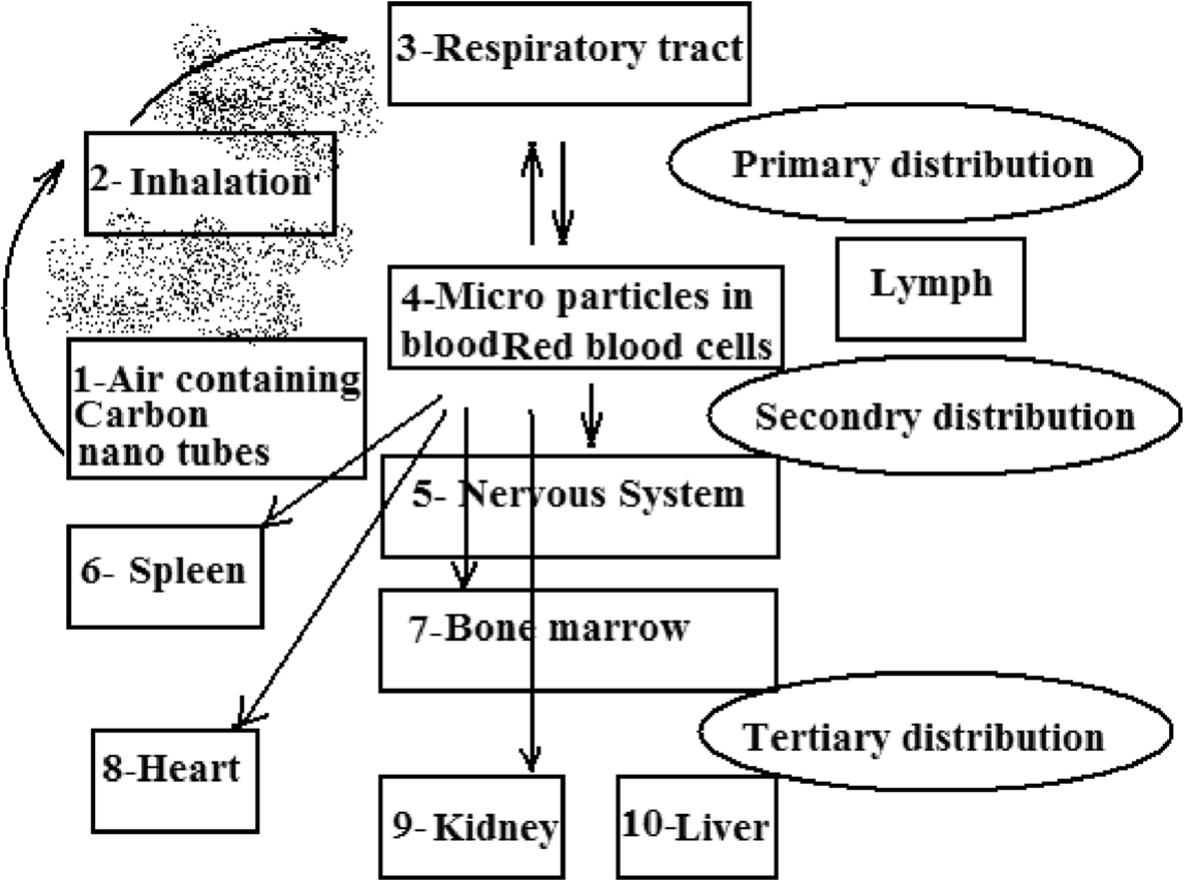
Distribution of CNTs in the body [69]

Several methods are available for the synthesis of nanotubes which are discussed in the synthesis section. For a wide application of CNTs, the most efficient method of synthesis is required, which can be scaled up to commercial production. Due to these reasons, arc-discharge and laser ablation synthesis methods are not a challenging technique due to difficulty in scaling up to commercial production. However, the CVD seems to be promising as it requires less expensive raw materials and utilizes the flowing gas as the carbon source, and less purification techniques are required for the purification. However, this technique requires more studies in terms of increasing yield and purity level. These methods produce both MWNTs as well as SWNTs depending on operating conditions. But still work can be done on the optimization of its operating condition for the bulk production of SWNTs at low cost [73]. The as-synthesized SWNTs consist of unwanted impurities like amorphous carbon, carbon particles, fullerenes, metal catalyst, etc. with the available methods [73]. These impurities affect the properties of nanotubes [1]. Many purification methods are available as discussed in the purification section; the commonly used methods are oxidation and acid refluxing. Oxidation method oxidizes the impurities as well as nanotubes, which decreases their properties [18]. However, the acid refluxing method only removes the metal catalyst leaving behind the nanotubes and carbon impurities which affect their properties [1]. Because of their unique properties, challenge can be taken to develop more methods of purification at low cost with high purity and good yield and with fewer defects of SWNTs. Carbon nanotubes have the ability to stick together in bundles [6], which decreases their unique mechanical properties or transport properties. Due to the strong surface interactions between the tubes, they produce phase separation on the failure of dispersion (Fig. 11). To avoid this, many commercial firms took the challenge of dispersing the CNTs and attempts are made through the use of ultra-sonication, extrusion, high shear mixing [64], etc., with little success. Increasing the ultra-sonication energy was found to improve the distribution of all CNT materials, but simultaneously, it decreases the size of nanotube ropes, enhancing the electrical conductivity and storage modulus. And it is reported that excessive energy minimizes the properties of composite CNTs (www.hielscher.com/ultrasonics/nano_03.htm). Another problem associated with the dispersion is that nanotubes suffer from insufficient bonding across the nanotube–matrix material interface [1]. Challenges can be taken to disperse nanotubes in the right way without minimizing its properties. Carbon nanotubes are becoming major concerns due to lack of knowledge on their toxicity. Since the nanotubes are not produced in large industries to emit smoke with which their toxicity to be predicted, and simultaneously due to their nano size, it is difficult to examine what happens when it is stored in open containers or exposed to the atmosphere or when it is inhaled. A study on the toxicity of CNTs proved that CNTs are toxic, and their toxicity depends on the properties of CNTs such as their structure, method of manufacturing, concentration, and dose [74]. Figure 12 explains the inhalation of CNTs affects the respiratory tract where their distribution will be more and also other parts of the body through the respiratory tract, which depends on the concentration and dose of CNTs. In vitro and in vivo studies have shown that those CNTs or their associated contaminants that are arising during the production may cause oxidative stress and prominent pulmonary inflammation [74]. This present scenario calls for immediate attention on standardization of packaging and handling of CNTs. For the companies and customer to compare the quality of different CNTs, the CNT industries require some form of standardization procedure. Due to lack of these standards, the companies and buyers are facing difficulties in assuring the safety of the product and process. Therefore, handling these materials by traditional methods increases the cost considerably. The limited knowledge on the safety of CNTs and their product misleads the public to believe the extent of the safety of these products. Hence, the standardization and regulation of these products are essential to bring the awareness in society for the commercial developments [23].

## Conclusions

Now, challenges can be taken to bring the awareness about the nanotubes in society by their standard and novel applications.
